# DNA Photocleavage in the Near-Infrared Wavelength Range by 2-Quinolinium Dicarbocyanine Dyes

**DOI:** 10.3390/molecules25122926

**Published:** 2020-06-25

**Authors:** Effibe O. Ahoulou, Kaitlyn K. Drinkard, Kanchan Basnet, Anna St. Lorenz, Oleh Taratula, Maged Henary, Kathryn B. Grant

**Affiliations:** 1 Department of Chemistry, Georgia State University, Atlanta, GA 30303, USA; eahoulou1@student.gsu.edu (E.O.A.); katiekiernan9322@gmail.com (K.K.D.); kbasnet1@student.gsu.edu (K.B.); 2 Department of Pharmaceutical Sciences, College of Pharmacy, Oregon State University, Portland, OR 97201, USA; lorenzan@oregonstate.edu (A.S.L.); oleh.taratula@oregonstate.edu (O.T.); 3 Center for Diagnostics and Therapeutics, Georgia State University, Atlanta, GA 30303, USA

**Keywords:** cyanine dyes, DNA strand scission, hydroxyl radicals, near-infrared photosensitizers, photodynamic therapy

## Abstract

Here, we report the syntheses of two pentamethine cyanine dyes containing quinolinium rings and substituted with either hydrogen (**3**) or bromine (**4**) at the *meso* carbon. The electron withdrawing bromine atom stabilizes dye **4** in aqueous buffer, allowing complex formation to occur between the dye and double-helical DNA. UV–visible, CD, and fluorescence spectra recorded at low DNA concentrations suggest that dye **4** initially binds to the DNA as a high-order aggregate. As the ratio of DNA to dye is increased, the aggregate is converted to monomeric and other low-order dye forms that interact with DNA in a non-intercalative fashion. The brominated dye **4** is relatively unreactive in the dark, but, under 707–759 nm illumination, generates hydroxyl radicals that cleave DNA in high yield (pH 7.0, 22 °C). Dye **4** is also taken up by ES2 ovarian carcinoma cells, where it is non-toxic under dark conditions. Upon irradiation of the ES2 cells at 694 nm, the brominated cyanine reduces cell viability from 100 ± 10% to 14 ± 1%. Our results suggest that 2-quinolinium-based carbocyanine dyes equipped with stabilizing electron withdrawing groups may have the potential to serve as sensitizing agents in long-wavelength phototherapeutic applications.

## 1. Introduction

Originally employed in photography to extend the wavelength range of silver halide emulsions, cyanine dyes are now extensively used in diverse fields ranging from biotechnology to laser technology [1,2,3]. Carbocyanines are typically composed of two nitrogen-containing heterocyclic aromatic rings that share a positive charge that is delocalized by the movement of electrons through a semi-flexible central polymethine bridge. These features make it possible for the monomeric and/or aggregated forms of many carbocyanine dyes to form stable complexes with double-helical DNA. Modes of cyanine binding, which include intercalation, groove binding, and external electrostatic interactions, depend upon the structure and aggregation state of the dye [4,5,6,7]. Many cyanines exhibit excellent optical properties that include high absorption coefficients, high fluorescence quantum yields, and absorption and emission maxima that are easily tuned by alternating terminal heterocyclic groups and by lengthening the dye’s central polymethine chain. For every two methine carbons added to the polymethine unit, absorption is red-shifted by ~100 nm [1]. This has made possible the design and synthesis of a variety of carbocyanine-based nucleic acid fluorescent sensors that absorb and emit light in the visible and/or near-infrared regions of the electromagnetic spectrum [1,8,9]. Near infrared excitation wavelengths from 700 to 900 nm are particularly attractive for biological applications because they are efficiently transmitted through biological tissue [10]. 

Low cellular toxicity coupled with the ability to absorb and emit near-infrared light has led to the development of indocyanine green (ICG; *λ*_ex_ = 800 nm; *λ*_em_ = 810 nm) and other cyanines as biological agents in the diagnosis and imaging of cancer [1,11,12,13,14]. The fluorophore ICG, approved by the United States Food and Drug Administration in 1956, is routinely used in medical diagnostics to monitor blood flow, and is now under investigation as a tumor-imaging agent. Although ICG and other cyanines are largely employed as fluorescent probes, it has been proposed that they should also be able to serve as effective phototherapeutic agents [12,15,16,17,18]. In photodynamic therapy (PDT), a light source is used to activate a photosensitizing agent (PS) in diseased tissue. This triggers the production of highly localized, short-lived reactive oxygen species (ROS) through the reaction of ground state triplet oxygen (^3^O_2_) with the photosensitizer’s triplet excited state (^3^PS*) [19,20,21]. Two major ROS are formed. Type 2 energy transfer gives rise to singlet oxygen (^1^O_2_), while in Type 1 electron transfer, superoxide anion radical (O_2_•^−^) formation generates highly reactive hydroxyl radicals (•OH) through an H_2_O_2_ intermediate [22,23]. With respective diffusion distances of 50–100 [24] and 0.8–6.0 nm [25], ^1^O_2_ and •OH cause highly localized, oxidative damage to DNA and other macromolecules within the irradiated cells [26]. This spatial targeting minimizes side effects, thereby enhancing positive clinical outcomes in the treatment of cancer and other diseases [27]. The clinically approved PDT agents porfimer sodium (Photofrin^®^), verteporfin (Visudyne^®^), and talaporfin (Aptocine™) all directly cleave genomic DNA in tissue culture and/or in circulating cells exposed to red light [20]. Because absorption of 700–900 nm irradiation by biological tissues is low, there is now great interest in the development of photosensitizing agents that are activated in the near-infrared wavelength range [10]. 

**Scheme 1:**
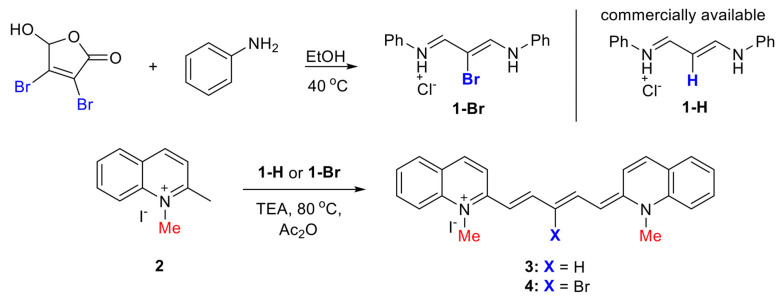
Syntheses of 2-quinolinium pentamethine carbocyanine dyes **3** and **4**.

Symmetrical benz[*e*]indolium [28] and 4-quinolinium [29] carbocyanine dyes as well as asymmetrical cyanines based on oxazole yellow (YO) and the YO sulfur analog thiazole orange (TO) [30,31,32,33] have been utilized to sensitize DNA photocleavage upon irradiation with visible and/or near-infrared light. Surprisingly, little is understood about the potential of other types of cyanine dyes to act as DNA photo-cleaving agents and even less is known about their phototherapeutic effects in cancer cells. Using a pentamethine linker to connect two 2-quinolinium rings, here we synthesized two symmetrical dicarbocyanine dyes that exhibit strong near infrared photo-nuclease activity (707 to 759 nm hν, pH 7.0; **3** and **4** in Scheme 1). In particular, *meso* brominated dye **4** photo-cleaves DNA in high yield and becomes highly cytotoxic to ES2 ovarian carcinoma cells when exposed to 694 nm illumination.

## 2. Results and Discussion

As shown in Scheme 1, the syntheses of the two pentamethine carbocyanine dyes **3** and **4** were conducted according to published synthetic procedures [28,34,35,36,37,38]. The 2-quinolinium rings were selected to promote DNA binding interactions [39] and a central pentamethine bridge was employed to red-shift dye absorption. In aqueous solutions, cyanine dyes, especially those with long polymethine chains, are predisposed to lose color over time due to spontaneous dye auto-oxidation (no hν) [40]. To render the dye a poorer reducing agent and thereby increase dye stability, the *meso* hydrogen atom in the pentamethine bridge of **3** was replaced with electron withdrawing bromine (**4**). The positioning of a halogen at the polymethine *meso*-carbon was also anticipated to introduce a “heavy atom effect” in which ROS production and DNA photocleavage might be enhanced by increasing the rate of intersystem crossing between the photosensitizer’s singlet and triplet excited states [41]. The brominated linker, **1**-**Br** (Scheme 1), was thereby created by gently heating mucobromic acid with aniline in dry ethanol until the starting material was consumed according to TLC [34]. The pentamethine quinolinium cyanine dyes were then synthesized by adding 2 equiv of the previously synthesized salt **2**, to either linker **1**-**H**, for dye **3**, or **1**-**Br**, to produce dye **4**. The two dyes **3** and **4** were precipitated with diethyl ether and recrystallized from methanol [28].

### 2.1. UV–Visible Spectrophotometry: Dye Stability and DNA Interactions

**Figure 1:**
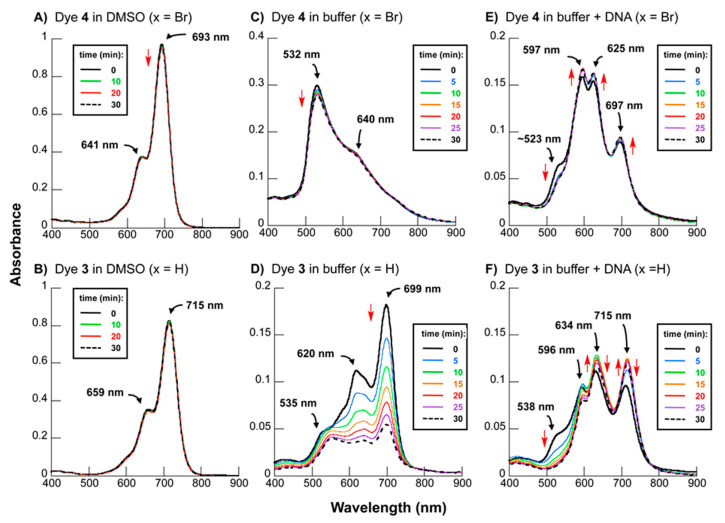
UV–visible spectra recorded over 30 min for 10 µM of dyes **4** and **3** in: (**A**,**B**) DMSO; (**C**,(**D**) 10 mM sodium phosphate buffer pH 7.0; and (**E**,**F**) 10 mM sodium phosphate buffer pH 7.0 and 150 µM bp CT DNA (22 °C).

In our first set of experiments, UV–visible spectra were recorded as a function of time. Dyes **4** and **3** were found to be stable in DMSO, displaying steady absorption at *λ*_max_ values of 693 (**4**, X = Br) and 715 nm (**3**, X = H) over 30 min (Figure 1A,B). When moved to aqueous buffer, the dyes’ 693- and 715-nm peaks were replaced by new blue-shifted maxima at 532 (**4**) and 699 nm (**3**) (Figure 1C,D). In contrast to DMSO, autooxidation of dye **3** (X = H) in the aqueous buffer was indicated by extensive absorption loss over time. Substitution of the *meso* hydrogen for electron withdrawing Br stabilized absorption, showing that dye **4** (X = Br) was considerably less susceptible to oxidation. Upon the addition of calf thymus (CT) DNA to the aqueous buffered solutions, the peak positions and intensities of dyes **4** and **3** were markedly altered. Both dyes were stabilized by the DNA, especially in the case of **4** (X = Br), which, in contrast to **3**, exhibited a net absorption gain over 30 min rather than a net absorption loss (Figure 1E,F).

### 2.2. DNA Photocleavage in the Near-Infrared Wavelength Range

**Figure 2:**
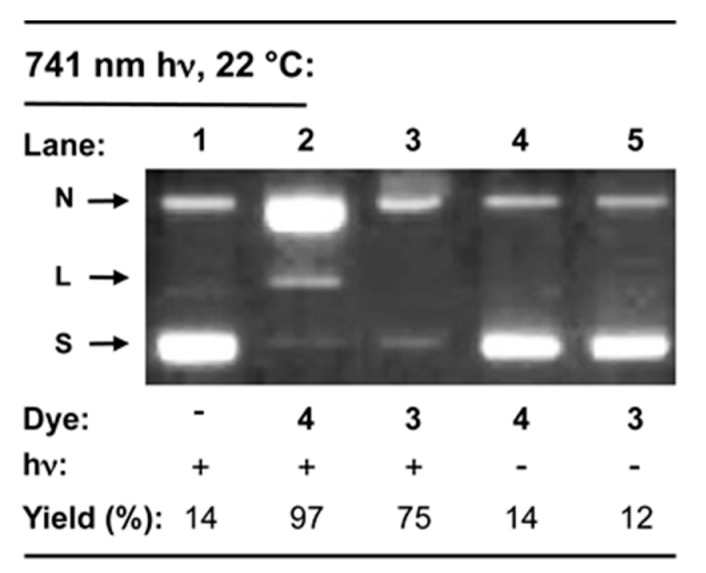
Ethidium bromide stained 1.5% agarose gel showing photo-cleavage of pUC19 plasmid DNA upon irradiation with a 741-nm LED lamp (0.3 W/cm^2^; spectral output 707–759 nm). Reactions contained 10 mM sodium phosphate buffer pH 7.0 and 38 µM bp DNA in the absence and presence of 25 µM of dyes **4** and **3** (60 min hν at 22 °C). Parallel control reactions containing dye were kept in the dark (lanes **4** and **5**). Abbreviations: L, linear; N, nicked; S, supercoiled.

Our next goal was to determine whether cyanine dyes **4** and **3** could act as photo-nucleases under physiological conditions of temperature and pH. With the development of phototherapeutic agents in mind, we employed a near-infrared 741-nm LED medical lamp (0.3 W/cm^2^) with a 707–759-nm spectral output overlapping DNA-bound dye absorption (Figure 1E,F). In a preliminary experiment, solutions containing 38 µM bp of pUC19 plasmid DNA in the absence and presence of 25 µM of dye were either irradiated for 60 min or kept in the dark (10 mM sodium phosphate buffer pH 7.0, 22 °C). Upon exposure to the near-infrared light, the reaction containing the brominated dye (**4**) generated very high levels of DNA strand scission (Lane 2 in Figure 2). Photocleavage yields were intermediate in the case of unstable H-substituted counterpart **3** (Lane 3) and low for negative control reactions that were either kept in the dark (Lanes 4 and 5 in Figure 2) or irradiated in the absence of dye (Lane 1). 

**Figure 3:**
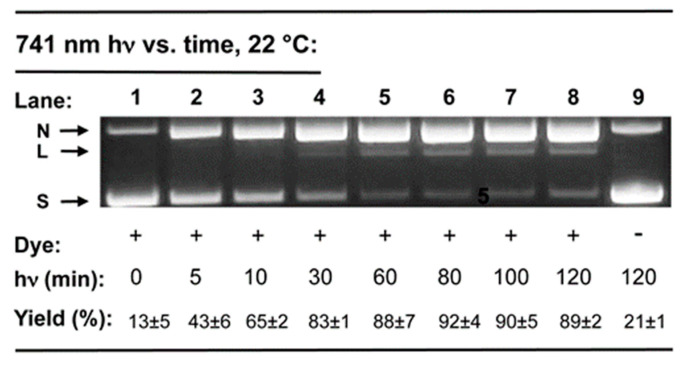
A representative agarose gel showing photo-cleavage as a function of time for 38 µM bp pUC19 DNA in 10 mM sodium phosphate buffer pH 7.0 (22 °C). Individual reactions in the presence and absence of 24 µM of dye **4** were irradiated with a 741-nm LED lamp (0.3 W/cm^2^; spectral output 707–759 nm) for time intervals ranging from 0 to 120 min. Data points are averaged over 2–3 trials. Errors represent standard deviation. Abbreviations: N, nicked; S, supercoiled.

**Figure 4:**
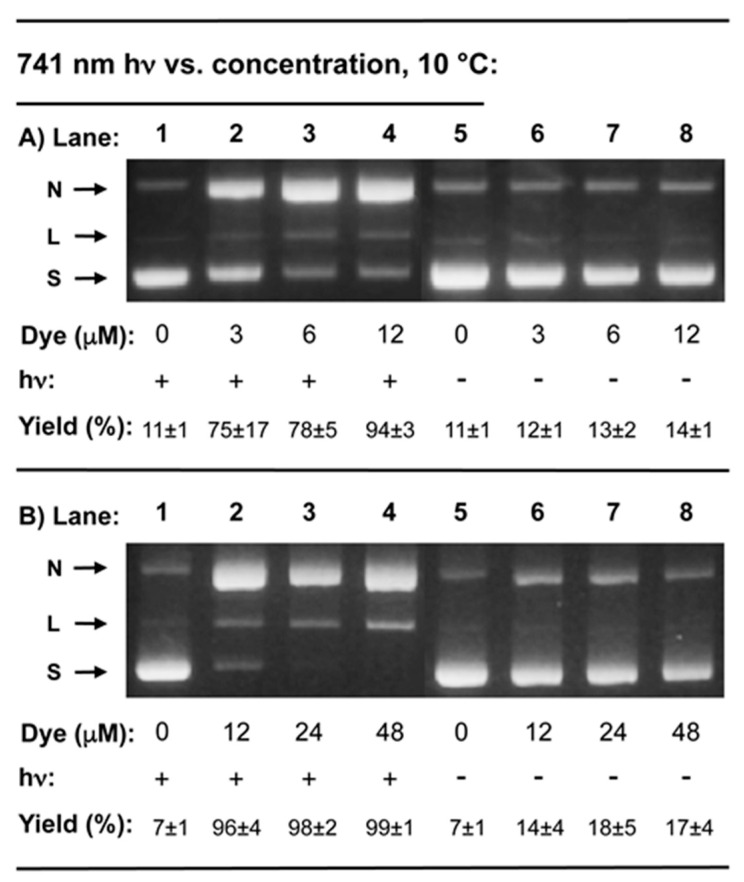
Representative agarose gels showing photocleavage of 38 µM bp pUC19 DNA in the presence of dye **4** concentrations ranging: (**A**) 0–12 µM; and (**B**) 0–48 µM (10 mM sodium phosphate buffer pH 7.0, 10 °C). Reactions were either irradiated with a 741-nm LED lamp (0.3 W/cm^2^; spectral output 707–759 nm) for 60 min or kept in the dark. Data points are averaged over 2–3 trials. Errors represent standard deviation. Abbreviations: N, nicked; S, supercoiled.

The superior stability and DNA-cleaving activity exhibited by brominated cyanine **4** encouraged us to study this dye further. (Cyanine **3** was no longer employed due to the propensity of this dye to degrade in aqueous solutions (Figure 1D,F).) In our next experiment, reactions containing 24 µM of **4** and 38 µM bp of pUC19 plasmid DNA were irradiated over intervals of time ranging from 0 to 120 min (10 mM sodium phosphate buffer pH 7.0, 22 °C; Figure 3). DNA cleavage was sensitized by the dye in a yield of 43 ± 6% after 5 min of irradiation and in increasing yield until approaching a plateau at ~60 min (88 ± 7% cleavage). We then lowered the reaction temperature from 22 °C to 10 °C and tested different amounts of dye (10 mM sodium phosphate buffer pH 7.0, hν 60 min; Figure 4). At the lower temperature, strong DNA photocleavage was observed. Yields ranged from 75 ± 17% for 3 µM of dye **4** up to 99 ± 1% at a dye concentration of 48 µM. 

### 2.3. Spectrophotometric Analyses of DNA Binding Modes

Aggregation of cyanines in aqueous solution typically involves the formation of cofacial *H*-aggregates with absorption maxima that are hypsochromic with respect to the cyanine monomer, and/or staggered cyanine J-aggregates that display bathochromic absorption [3]. The monomeric as well as aggregated carbocyanines can bind to DNA in minor groove as well as externally, depending on the structure of the dye [6,7,29,42,43]. Intercalative DNA binding is possible for monomeric cyanines, but is highly unlikely in the case of the H and J forms due to self-stacking of the dyes’ aromatic rings within the aggregate [3]. To better understand how aggregation effects might be influencing DNA interactions, additional spectroscopic measurements of brominated cyanine dye **4** were carried out.

#### 2.3.1. UV–Visible Absorption Spectrophotometry

**Figure 5:**
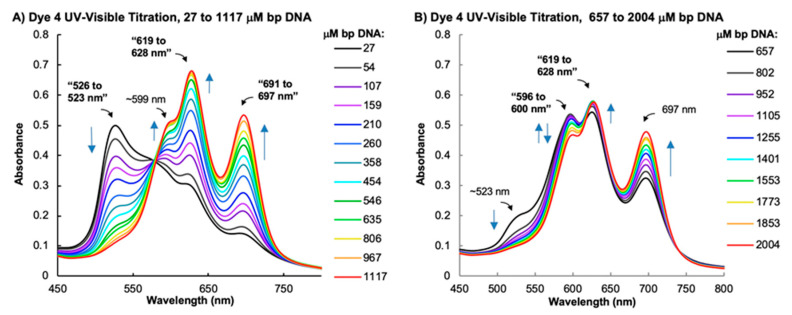
UV–visible absorption titration spectra at 22 °C of 20 µM of dye **4** in the presence of 10 mM sodium phosphate buffer pH 7.0 and CT DNA concentrations ranging: (**A**) 27–1117 µM bp; and (**B**) 657–2004 µM bp. All absorption spectra are corrected for sample dilution.

In our first experiment, absorption titrations were conducted in which small amounts of CT DNA titrant were sequentially added to a solution containing a fixed amount of dye **4** (Figure 5). Raising DNA concentration is known to disrupt cyanine aggregation in favor of the formation monomeric dye [5,7,42]. These changes in aggregation state are often reflected in the absorption spectra of the dye. This being said, the introduction of CT DNA to **4** produced major cyanine peak positions with *λ*_max_ values that varied as a function of increasing DNA concentration. In the case of the dye’s lowest wavelength peak position at 526 nm, sequential DNA addition gradually shifted the *λ*_max_ from 526 to 523 nm while significantly decreasing absorption. At the same time, the intensities increased in the case of two major bathochromic peaks with respective *λ*_max_ values that changed from 619 to 628 nm and from 691 to 697 nm (Figure 5A). In DMSO, a polar organic solvent that promotes monomer formation [6,29,44], dye **4** absorbs strongly at 693 nm (Figure 1A). When moved to buffered water, a highly polar solvent that supports dye aggregation, the 693-nm peak was no longer observed and was replaced by a major absorption band at 532 nm (no DNA; Figure 1C). These effects suggest that the hypsochromic “526–523-nm” feature in the absorption titration shown in Figure 5A represents either free or DNA-bound *H*-aggregated dye, while the “691–697-nm” peak corresponds to a DNA-bound dye monomer. At very high DNA concentrations (Figure 5B), the height of a ~599 nm shoulder in the absorption titration spectra in Figure 5A became a more prominent spectral feature with λ_max_ values that ranged from 596 to 600 nm. The absorption of this “596–600-nm” band in the high DNA concentration range initially increased but then substantially decreased relative to a concomitant net increase in “619–628-nm” absorption (Figure 5B). These trends suggest that the “596–600-nm” and “619–628-nm” spectral features in the absorption titrations shown in Figure 5B represent two distinct DNA bound dye species, possibly dimers or low-order aggregates.

#### 2.3.2. Circular Dichroism

**Figure 6:**
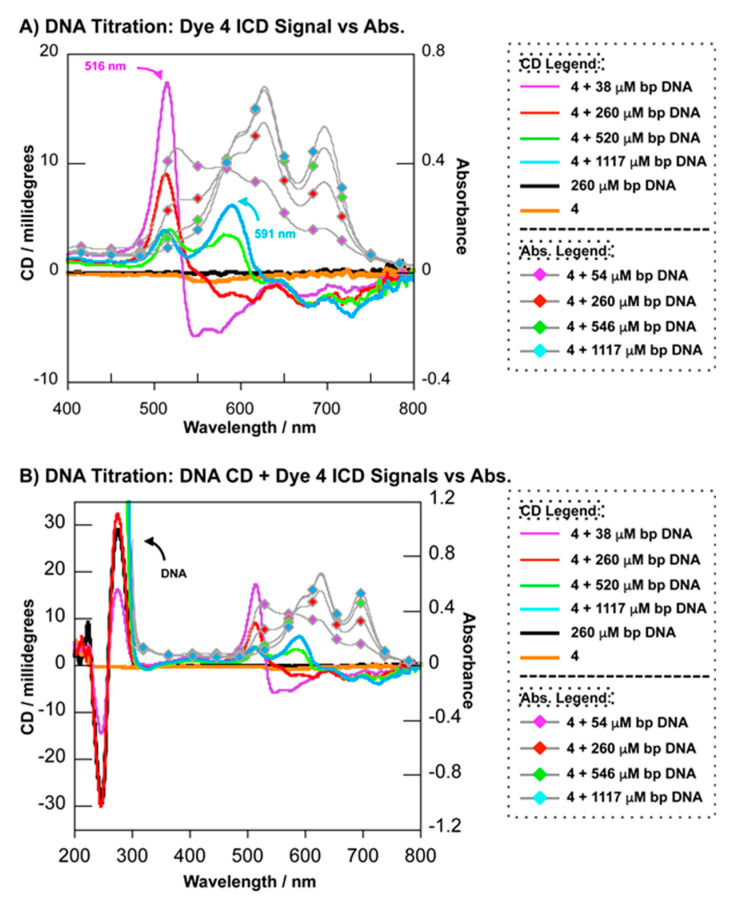
Double *y*-axis plots superimposing CD titration spectra (showing DNA CD and dye **4** induced circular dichroism (ICD) signals) vs. corresponding UV–visible absorption (Abs.) titration spectra (22 °C). Samples contained 10 mM sodium phosphate buffer pH 7.0 in the presence of 20 µM of dye **4** and/or CT DNA. Absorption spectra are from Figure 5A. (**A**): CD and absorption spectra are plotted from 400 to 800 nm. (**B**): The CD and absorption data in (**A**) re-plotted from 200 to 800 nm and 300 to 800 nm, respectively.

Circular dichroism (CD) represents a convenient and informative tool for gaining knowledge of cyanine dye/DNA interactions. Binding of an achiral cyanine to chiral B-form DNA typically generates an induced circular dichroism (ICD) signal that can reveal information about the dye’s DNA binding mode and aggregation state. For example, a bisignate exciton ICD signal is usually indicative of the formation of groove bound [6,7,42] or externally bound [43] high- and low-order aggregates as well as adjacent end-to-end cyanine dimers. In contrast, individual groove-bound monomers and groove-bound dimers do not exhibit exciton coupling and instead tend to generate positive ICD signals [7,45]. The ICDs of cyanine intercalators are relatively weak and are either positive [45] or negative [5,43,46], depending on the orientation of the transition moment of the dye relative to the dyad axis of the DNA duplex [47]. This being said, we recorded circular dichroism spectra of brominated dye **4** as a function of increasing CT DNA concentration. The ICD signals generated by binding of the achiral cyanine to the chiral DNA were then plotted against corresponding absorption spectra shown in Figure 5A (Figure 6A). As expected, dye **4** had no CD signal in the absence of the CT DNA. When the DNA was introduced, however, the CD titration data revealed the formation of an apparent bisignate ICD band indicative of exciton coupling interactions between DNA-bound dye molecules. This bisignate feature is comprised of a positive short wavelength component at 516 nm and a negative long wavelength component that appears to be partially attenuated by a positive ICD signal at 591 nm. The central position of the bisignate band and its intensity as a function of DNA concentration correspond to the hypsochromic “526–523-nm” absorption feature we tentatively attributed to the formation of an *H*-aggregate (Figure 5A and Figure 6A). Typically, *H*-aggregated cyanines bind in the DNA minor groove through the formation of cofacial cyanine dimers that assemble in right-handed, end-to-end fashion that matches the right-handed curvature of B-form duplex DNA [6,7,48]. The hypsochromic “526–523-nm” absorption peak in Figure 5A and Figure 6A could indeed represent an *H*-aggregate. However, the ordering of the two components the ICD band, with the positive peak at shorter wavelengths and negative peak at longer wavelengths, points to a left-handed orientation that is inconsistent with the formation a groove-bound complex involving right-handed B-form DNA [6,7]. The bisignate DNA CD signal in the extended CD spectra shown in Figure 6B confirms that the nucleic acid maintains a right-handed B-form helical shape upon complex formation with dye **4**. We therefore conclude that the peak ordering in the bisignate cyanine ICD band suggests that the “526–523-nm” aggregate may be bound to DNA in an atypical fashion rather than in one of the DNA grooves [43]. In addition to the dye’s bisignate ICD, the second prominent feature of the CD titration in Figure 6A is the overlapping positive ICD signal at 591 nm. This peak is closest in position to the “596–600-nm” absorption feature in the high DNA concentration UV–visible titration (Figure 5B). Assuming that there is no hidden exciton coupling, the intensity and sign of the 591-nm ICD point to the formation of a cyanine dimer. If exciton coupling is being obscured by overlap with one or more adjacent ICD signals, then a low-order aggregate associated with either the “596–600-nm” or the “619–628-nm” absorption feature might be indicated.

#### 2.3.3. Fluorescence Spectroscopy

**Figure 7:**
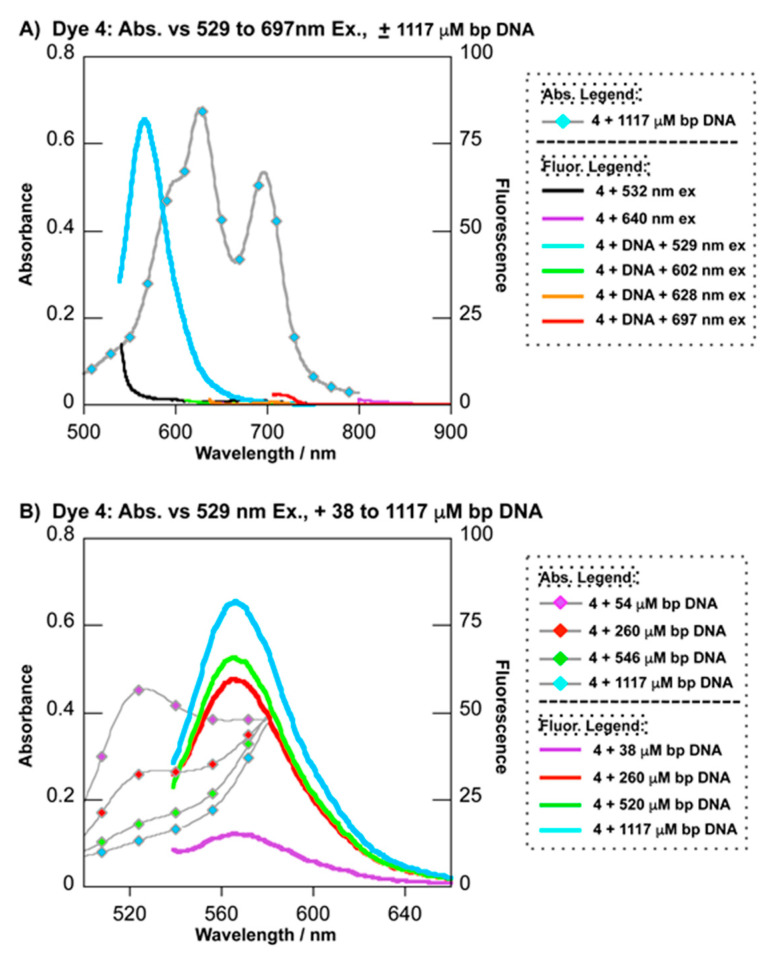
Double *y*-axis plots superimposing dye **4** fluorescence emission spectra (Em) with dye **4** UV–visible absorption (Abs) (22 °C). Samples contained 10 mM sodium phosphate buffer pH 7.0, 10 µM of dye (Em) or 20 µM of dye (Abs) and: (**A**) 1117 µM bp of CT DNA, where the emission spectra were recorded at the excitation wavelengths (Ex) 532 and 640 nm (dye **4**); 529, 602, 628, and 697 nm (dye **4** with DNA); and (**B**) 38–1117 µM bp of CT DNA, where the emission spectra were recorded at an excitation wavelength (Ex) of 529 nm. The absorption spectra are from Figure 5A and in (**B**) are re-plotted from 500 to 580 nm for clarity.

Figure 7A superimposes the absorption spectrum of dye **4** in the presence of 1117 µM bp of CT DNA with fluorescence emission that was generated at excitation wavelengths that overlap the major absorption features of the free (*λ*_ex_ = 532 and 640 nm; Figure 1C) and DNA-bound dye (*λ*_ex_ = 529, 602, 628, and 697 nm; Figure 5). Dye **4** did not fluoresce when the CT DNA was absent (Figure 7A). When 1117 µM bp of CT DNA was added to the solution, however, emission was observed only at the *λ*_ex_ = 529 nm wavelength (blue line in Figure 7A). Interestingly, the corresponding “526–523-nm” absorption feature overlapping this wavelength was of very low intensity and almost completely obscured by other dye absorption features (Figure 7A). Based on the UV–visible absorption and ICD results from the preceding two sections, we had tentatively assigned the “526–523-nm” feature to be that of an atypical, high-order dye aggregate that is converted to monomeric and other low-order dye forms as DNA concentration is increased. 

In our next set of fluorescence measurements, we excited dye **4** at 529 nm over a range of CT DNA concentrations and superimposed the resulting emission spectra with corresponding absorption spectra from Figure 5A (Figure 7B). Interestingly, as absorption by the “526–523-nm” DNA bound high-order aggregate increased at the low DNA concentrations, the emission intensity upon 529-nm excitation decreased in an inversely proportional fashion. This suggests that the putative aggregate might be quenching its own fluorescence at high aggregate concentrations. Fluorescence self-quenching of aggregated dyes is a well-known phenomenon that can arise from close packing of dye molecules and is a common occurrence for even widely used fluorophores such as fluorescein and rhodamine [49,50]. Our results indicate that “diluting” the aggregate by adding DNA lowers its concentration relative to other dye forms. This may partially reverse the self-quenching process, allowing aggregate fluorescence to increase (Figure 7B) [50].

Strong cyanine dye fluorescence is typically observed when the conformational mobility causing rapid non-radiative decay from the singlet excited state of free dye is restricted, i.e., by intercalative DNA binding [3,5]. While dimeric and aggregated cyanines cannot intercalate, the absence of a fluorescence signal in the case of the “691–697-nm” putative DNA-bound monomer would seem to rule against its ability to interact with DNA by intercalation. The “526–523-nm” high-order aggregate is fluorescent at low dye concentrations, suggesting that there is less flexibility in its structure compared to the non-fluorescent “596–600-nm” and “619–628-nm” low-order cyanine forms.

#### 2.3.4. Competitive DNA Binding

**Figure 8:**
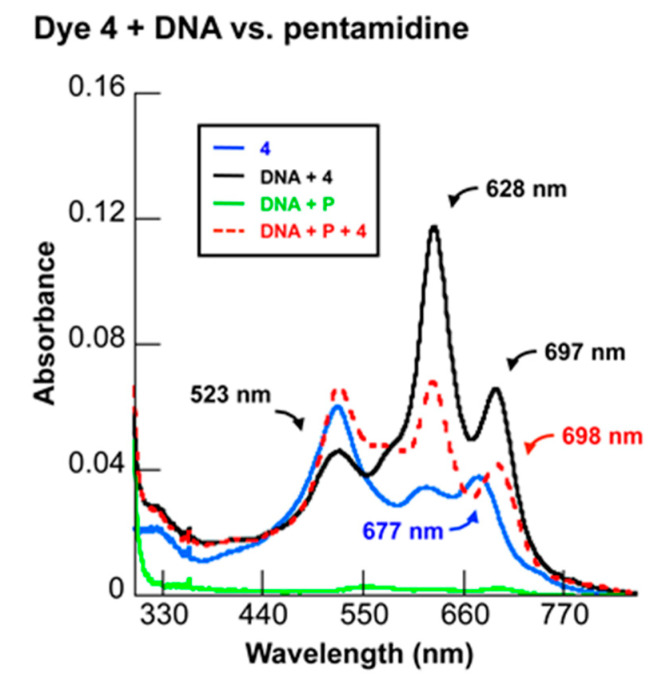
UV–visible absorption spectra recorded for: dye **4** without CT DNA (blue lines); dye **4** with CT DNA (black lines); pentamidine (P) with CT DNA (green lines); and dye **4** with DNA pre-equilibrated for one min with pentamidine (red lines). All samples contained 10 mM sodium phosphate buffer pH 7.0 and one or more of the following: 10 µM of dye **4**, 150 μM bp of CT DNA, and 10 µM of pentamidine.

In our next experiment, UV–visible spectrophotometry was used to compare aqueous solutions of dye **4** without and with untreated CT DNA vs. solutions containing a CT DNA preparation that had been pre-equilibrated with the known minor groove binder pentamidine. The results of the pentamidine pre-treatment are shown in Figure 8. Addition of the pentamidine increased the ratio of aggregated “526–523-nm” dye absorption relative to the putative low-order and monomeric dye forms absorbing at “619–628-nm” and “691–697-nm”, respectively. Unlike ethidium bromide, which unwinds and lengthens DNA [51], pentamidine makes minimal changes to DNA duplex structure [52,53]. Taking this into consideration, the experimental results suggest that pentamidine might be reducing the number of dye-accessible minor groove binding sites on the DNA. This could conceivably prevent the “619–628-nm” low-order and “691–697-nm” monomeric dye forms from binding to DNA in the minor groove and at the same time might promote the formation externally bound “526–523-nm” high-order aggregate.

### 2.4. Mechanistic Analysis of Dye-Sensitized DNA Photocleavage

**Scheme 2:**
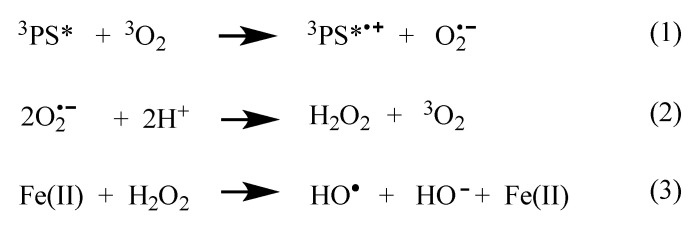
Possible mechanism for accounting for dye **4**-sensitized hydroxyl radical formation [20,21].

Experiments to probe for cyanine dye-sensitized ROS production were conducted using chemical additives to alter DNA direct strand break formation. Irradiating plasmid DNA reactions in the presence of the •OH scavenger sodium benzoate reduced strand scission by 77 ± 9%, strongly implicating the involvement of Type 1 hydroxyl radicals in dye **4**-sensitized DNA cleavage (Table 1 and Figure S1 in the Supplementary Materials). The metal ion chelating agent EDTA and the hydrogen peroxide reducing enzyme catalase decreased photocleavage yields by ~73%, and ~30%, respectively, pointing to Fenton chemistry as a possible hydroxyl radical source. This being said, the mechanism shown in Scheme 2 can be considered. In this series of reactions, the photosensitizer triplet state of dye **4** reduces ground state triplet oxygen ^3^O_2_ by Type 1 electron transfer to yield superoxide anion radicals (O_2_•^−^) (Line 1). Then, spontaneous dismutation of the O_2_•^−^ gives rise to H_2_O_2_, which, in the presence of trace Fe(II), triggers the production of hydroxyl radicals (•OH) by the Fenton reaction (Lines 2 and 3) [22,23]. To test for the participation of an alternative ROS pathway involving DNA damage by Type 2 singlet oxygen (^1^O_2_), we replaced H_2_O with D_2_O, a solvent that produces a 10-fold enhancement in singlet oxygen lifetime [54]. Because levels of dye **4** sensitized DNA strand scission were suppressed by the D_2_O rather than undergoing an enhancement, we concluded that it is unlikely that Type 2 singlet oxygen makes a major contribution to dye **4**-sensitized DNA photocleavage.

### 2.5. Uptake, Distribution, and Phototoxicity of Dye **4** in Ovarian Cancer Cells

**Figure 9:**
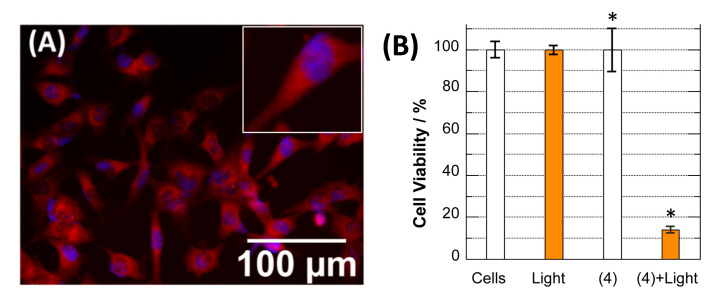
(**A**) A representative superimposed fluorescence microscopy image reveals intracellular dye localization (red) in ES-2 ovarian clear cell adenocarcinoma cells after incubation with 0.5 μg/mL of dye **4** for 24 h followed by staining nuclei with Hoechst 33342 (blue). Inset: The enlarged area of one cell within the fluorescence microscopy image. (**B**) ES2 cancer cell viability for: Cells, no treatment; Light, cells exposed to a 694-nm laser (~1.3 W/cm^2^) for 5 min; (**4**), cells incubated with dye **4** (0.5 μg/mL = 0.9 μM) for 24 h under dark conditions; and (**4**) + Light, cells incubated with dye **4** (0.5 μg/mL) for 24 h and exposed to a 694-nm laser (1.3 W/cm^2^) for 5 min. * *p* < 0.05 when compared with non-treated cells. Error bars represent standard deviation.

The cellular internalization efficiency of dye **4** was validated in fluorescence microscopy studies [55]. After incubation of the dye with ES2 ovarian carcinoma cells [56], the intrinsic NIR fluorescence signal of **4** was visible universally throughout the population of cells within their intracellular cytosolic regions. There was a definitive perinuclearly localization around the Hoescht nuclear stain as well as some overlap indicating possible nuclear uptake (Figure 9A). A Calcein AM assay was then used to assess the viability of the cells after treatment with 0.5 μg/mL of the dye in the dark and under light exposure [57]. Our results reveal that, although **4** was non-toxic in the dark at the tested concentration (0.5 μg/mL), the dye was capable of exhibiting substantial phototoxicity to the ES2 cancer cells when exposed to 694-nm light illumination (Figure 9B). Of note, the synthesized dye had low dark toxicity (Figure S2), with an IC_50_ value of 72.1 μg/mL.

## 3. Materials and Methods

### 3.1. Synthesis

#### 3.1.1. General

All chemicals and solvents were of American Chemical Society grade or HPLC purity and were used as received. HPLC grade acetonitrile (CH_3_CN) and water were purchased from VWR International (West Chester, PA, USA) and American Bioanalytic (Natick, MA, USA), respectively. All other chemicals were from Fisher Scientific (Pittsburgh, PA, USA) and Sigma-Aldrich (Saint Louis, MO, USA). Melting points (mp, open Pyrex capillary) were measured on a Meltemp apparatus and are uncorrected. ^1^H NMR spectra were recorded on a BrukerAvance (400 MHz) spectrometer (Bruker Corporation, Billerica, MA, USA). Chemical purity was also confirmed using ultra-performance liquid chromatography (UPLC, Waters, Milford, MA, USA) combined with simultaneous evaporative light scattering detection (ELSD), absorbance (photodiode array; PDA), fluorescence, and electrospray time-of-flight (ES-TOF) mass spectrometry (MS) (Waters, Milford, MA, USA).

#### 3.1.2. Procedure for the Preparation of Dyes **3** and **4**

Known compounds **3** and **4** were prepared based on the corresponding published synthetic procedures [28,34,35,36,37,38]. A mixture of salt **2** (2 equiv); malonaldehyde bisphenylimine monohydrochloride, **1**-**H**, or the brominated **1**-**Br** analog (1 equiv); and triethylamine (3 equiv) were refluxed for 3 h in acetic anhydride and then cooled to room temperature. The crude products were precipitated by adding 5 mL of diethyl ether or ethyl acetate to the reaction mixture. Then compounds **3** and **4** were precipitated, filtered, and dried [28,34]. The final products of dyes **3** and **4** were obtained as pure by crystallization from methanol.

*1-methyl-2-((1E,3E)-5-((E)-1-methylquinolin-2(1H)-ylidene)penta-1,3-dien-1-yl)quinolin-1-ium iodide;* (**3**); A green powder (56%). ^1^H NMR (400 MHz, DMSO-*d*6) δ ppm 3.88 (s, 6 H), 6.38 (d, *J* = 12.8 Hz, 2 H), 6.58 (t, *J* = 11.2 Hz, 1 H), 7.43 (m, 2 H), 7.71–7.74 (m, 2 H), 7.79–7.85 (m, 6 H), 7.97–8.04 (m, 4 H) (Figure S3).

*2-((1E,3Z)-3-bromo-5-((E)-1-methylquinolin-2(1H)-ylidene)penta-1,3-dien-1-yl)-1-methyl-quinolin-1-ium iodide;* (**4**); A green powder (49%). ^1^H NMR (400 MHz, DMSO-*d*6) δ ppm 4.00 (s, 6H), 6.32 (d, *J* = 13.2 Hz, 2 H), 7.53 (t, *J* = 7.6 Hz, 2 H), 7.81 (t, *J* = 7.6 Hz, 2 H), 7.90–7.94 (m, 4H), 8.02 (d, *J* = 8.8 Hz, 2 H), 8.22 (d, *J* = 9.6 Hz, 2 H), 8.34 (d, *J* = 13.2 Hz, 2 H) (Figure S4).

### 3.2. Analysis of Dye-DNA Interactions

#### 3.2.1. General

Deionized distilled water was used for the preparation of all aqueous solutions. Standard laboratory protocols were employed to clone pUC19 plasmid DNA in XL-1 blue *E. coli* competent cells (Stratagene, San Diego, CA, USA) [58]. The cloned plasmid was then isolated and purified using a QIAfilter Plasmid Mega Kit according to manufacturer’s instructions (Qiagen™, Hilden, Germany; Cat. No. 12263). Sodium phosphate buffer pH 7.0 used in DNA solutions was prepared from sodium phosphate monobasic and sodium phosphate dibasic salts (Thermo Fisher Scientific, Waltham, MA, USA). Sonicated calf thymus (CT) DNA was purchased from Invitrogen (Carlsbad, CA, USA; 10 mg/mL, average size ≤ 2000 bp, Cat. No. 15633-019), while deuterium oxide (99.9%) and agarose were, respectively, obtained from Cambridge Isotope Laboratories and BioRad. All other reagents, including sodium benzoate (99%), dimethyl sulfoxide (DMSO, ≥99.99%), ethidium bromide, pentamidine isethionate salt, and bovine liver catalase were supplied by Sigma-Aldrich.

UV–visible absorption spectra and fluorescence emission spectra were respectively acquired using PerkinElmer Lambda 35 (PerkinElmer, Waltham, MA, USA) and a PerkinElmer LS55 spectrophotometers (PerkinElmer, Waltham, MA, USA). Either a Jasco J-810 (Jasco, Easton, MD, USA) or a Jasco J-1500 CD spectropolarimeter (Jasco, Easton, MD, USA) was employed to record CD and ICD spectra.

#### 3.2.2. DNA Photocleavage

Thirty microliter samples consisting of 3–48 µM of cyanine dye, 38 µM bp of pUC19 plasmid, and 10 mM of sodium phosphate pH 7.0 were either kept in the dark or irradiated from 5 to 120 min with a near-infrared 741-nm LED medical lamp (0.3 W/cm^2^, 707–759-nm spectral output; Larson Electronics, Kemp, TX, USA). Reaction temperature was maintained at 10 or 22 °C by placing the samples in a thermometer-fitted metal block that was heated, kept at room temperature, or immersed in an ice bath during the reaction. In total, 3 µL of electrophoresis loading buffer containing 15.0% (*w*/*v*) ficoll and 0.025% (*w*/*v*) bromophenol blue was added to each sample and 20 µL of the resulting solution were transferred to the wells of 1.5% agarose gel stained with ethidium bromide (0.5 µg/mL, final concentration). Completely loaded gels were electrophoresed at 100 V for 1 h in a Bio-Rad Laboratories gel box containing 1× tris-acetate-EDTA (TAE) as running buffer and 0.5 µg/mL ethidium bromide. The gels were then visualized at 302 nm with a VWR Scientific LM-20E transilluminator, photographed with a UVP PhotoDoc-It™ Imaging System, and quantitated using ImageJ software. In the case of supercoiled DNA, integrated numerical values were multiplied by a correction factor of 1.22 to account for the decreased affinity of ethidium bromide for supercoiled vs. nicked and linear plasmid forms. The DNA photocleavage yields were then calculated according to the formula:

Percent Photocleavage = [(Linear + Nicked DNA)/(Linear + Nicked + Supercoiled DNA)] × 100.

#### 3.2.3. UV–Visible Spectrophotometry

Quartz cuvettes contained either 10 μM of cyanine dye in DMSO or 10 μM of dye in 10 mM of sodium phosphate pH 7.0 buffer without and with 150 µM bp CT DNA. Absorption spectra were recorded at 5-min time intervals from 0 to 30 min (22 °C).

In DNA titration experiments, small volumes of a concentrated aqueous CT DNA solution were successively added to solutions containing 20 μM of cyanine dye **4** in 10 mM sodium phosphate buffer pH 7.0 (500 μL initial volume). After spectra were acquired, absorption readings were corrected for sample dilution. Final CT DNA concentrations ranged from 0 to 2004 μM bp.

In competitive binding experiments, samples contained 10 μM of dye **4** in 10 mM of sodium phosphate pH 7.0 buffer without and with 150 µM bp CT DNA or 150 µM bp CT DNA pre-equilibrated with 10 μM of pentamidine for one min.

#### 3.2.4. Circular Dichroism

Individual CD samples consisted of 10 mM sodium phosphate buffer pH 7.0 with 10 µM of dye and 38–1117 µM of CT-DNA present alone and in combination (2000 µL total volume). Spectra were collected from 800 to 200 nm in 3 mL (1.0 cm) quartz cuvettes (Starna, Atascadero, CA, USA) using the following instrument settings: scan speed, 100 nm/min; response time, 2 s; bandwidth, 0.5 nm; and sensitivity, 100 millidegrees. The temperature was kept constant at 22 °C. Final spectra were averaged over 12 acquisitions.

#### 3.2.5. Fluorescence Spectroscopy

Solutions containing 10 mM sodium phosphate buffer pH 7.0 and 10 μM of cyanine dye **4** in the absence and presence of 38–1117 µM of bp of CT DNA were transferred to 3.0 mL Starna quartz cuvettes (2000 μL, total volume). The samples were excited at an appropriate wavelength ranging from 529 to 697 nm and emission spectra were recorded (22 °C). The scan speed of the spectrophotometer was 100 nm/min, gain was set at medium, and the excitation and emission slit widths were 4.5 nm.

#### 3.2.6. Regent-Induced Changes in DNA Photocleavage

Reactions containing 10 mM of sodium phosphate buffer pH 7.0, 38 µM bp pUC19 plasmid DNA and 24 µM of dye **4** were prepared in the presence and absence of 100 mM of sodium benzoate, 100 U/µL of catalase, 100 mM EDTA, or 72% D_2_O (*v*/*v*). The reactions were irradiated for 60 min (707–759 nm), resolved on 1.5% non-denaturing agarose gels, visualized, and quantitated as just described. The percent change in DNA photocleavage was calculated using the following formula, where the additive was either sodium benzoate, catalase, EDTA, or D_2_O:

Percent Change in Cleavage = [(% Total of Linear and Nicked DNA _with additive_ – % Total of Linear and Nicked DNA _without additive_)/(% Total of Linear and Nicked DNA _without additive_)] × 100.

### 3.3. Cytotoxicity Studies

#### 3.3.1. Cell Line

ES2 human clear cell ovarian carcinoma cell line was obtained from ATCC (Manassas, VA, USA). All cancer cells were cultured in DMEM medium (Sigma, St. Louis, MO, USA) with 10% fetal bovine serum and 1% penicillin–streptomycin (VWR, Visalia, CA, USA). All cells were grown in a humidified atmosphere of 5% CO_2_ (*v*/*v*) in air at 37 °C [56].

#### 3.3.2. Cellular Uptake and Fluorescence Imaging

ES2 cells were plated in 96-well plates at a density of 10 × 10^3^ cells/well and cultured for 24 h. After that, cells were incubated for 24 h in complete DMEM media containing dye **4** (0.5 μg/mL) dissolved in DMSO (<1%). Cells were then washed with DPBS, stained with Hoechst 33342 [29] and the subcellular distribution of the dye was imaged with an BZ-X710 Keyence microscope using DAPI filter and Cy^®^ 7 filter cubes [55].

#### 3.3.3. Evaluation of Phototherapeutic Effect

ES2 cells were plated in 96-well plates at a density of 10 × 10^3^ cells/well and cultured for 24 h. After that, the cells were incubated in the dark for 24 h in complete DMEM media containing dye **4** (0.5 μg/mL) dissolved in DMSO (<1%). The dye-containing media was then removed, and the cells were rinsed and left in DPBS while being exposed to a 694-nm laser diode light for 5 min (1.3 W/cm^2^). After treatment, cells were cultured for 24 h in complete DMEM growth medium prior to viability measurements with Calcein AM as previously described [57]. Non-treated cells, cells incubated with the same concentration of the dye **4** under dark conditions, and non-treated cells exposed to a 694-nm laser diode for 5 min were used as controls.

## 4. Summary and Conclusions

In photodynamic therapy, an ideal DNA photosensitizer should function optimally within a near-infrared phototherapeutic therapeutic window that affords maximum penetration depth of incident light through biological tissue [10]. Here, we showed that symmetrical 2-quinolinium pentamethine carbocyanine dyes substituted with hydrogen (**3**) or bromine (**4**) generate DNA cleavage when irradiated in the near-infrared wavelength range. The electron withdrawing bromine atom promotes DNA interactions by substantially reducing dye autooxidation in aqueous solution. UV–visible, circular dichroism, and fluorescence spectra of the brominated 2-quinolinium pentamethine cyanine (**4**) show that DNA has a major effect on dye aggregation. Low DNA concentrations favor the formation of a high-order hypsochromic cyanine aggregate. As DNA concentration is gradually increased, however, the dye **4** aggregate is sequentially converted to a bathochromic dye monomer through intermediate absorbing dimeric and/or low-order aggregated species. The high-order aggregate appears to interact with DNA in an external fashion, while the monomeric and low-order forms of dye **4** may be associating with DNA via its minor groove. The spectroscopic data also suggest that intercalative interactions do not play a major role in DNA binding. Photocleavage reactions run in the presence and absence of radical scavenging agents and other chemical additives reveal that irradiation of the monomeric, DNA-bound form of dye **4** with 707–759-nm near–infrared light generates Type 1 •OH radicals that produce direct strand breaks in plasmid DNA in high yield (pH 7.0, 22 °C). Using fluorescence microscopy, we were able to show that the dye is readily taken up by ES-2 cells where it localizes in the intracellular cytosolic and perinuclear regions and possibly in the cell nuclei. While non-toxic in the dark, brominated cyanine **4** displayed high levels of phototoxicity over six trials, reducing ES-2 cell viability from 100 ± 10% to 14 ± 1% when irradiated with a 694-nm laser. We will continue to explore and design new 2-quinolinium dicarbocyanine dyes with the aim of developing optimal DNA photosensitizing agents for near-infrared phototherapeutic applications.

## Figures and Tables

**Table 1:** Inhibition of dye **4** photocleavage induced by chemical additives ^1^.

Reagents Added	Target	Cleavage Inhibition (%)
Na benzoate	•OH	77 ± 9
D_2_O	^1^O_2_	11 ± 0.4
Catalase	H_2_O_2_	30 ± 10
EDTA	Fe^2+^, Fe^3+^/Cu^1+^, Cu^2+^	73 ± 8

^1^ Reactions consisting of 38 µM bp of pUC19 plasmid DNA equilibrated with 24 µM of **4** with and without 100 mM sodium benzoate or EDTA, 100 U/µL of catalase, or 72% D_2_O (*v*/*v*) were irradiated for 60 min with a 741-nm LED lamp (0.3 W/cm^2^, spectral output 707–759 nm; 10 mM sodium phosphate buffer pH 7.0). Data were averaged over 2–3 trials with error reported as standard deviation.

## Supplementary Materials

The following items are available online, Figure S1: Dye **4**-sensitized near-infrared DNA photocleavage gels in the presence of chemical additives; Figure S2: Dye **4** toxicity against ES2 cells under dark conditions; Figure S3: ^1^H NMR spectrum of dye **3**; Figure S4: ^1^H NMR spectrum of dye **4**.

## Abbreviations

The following abbreviations are used in the manuscript:

bp	base pair
CT DNA	calf thymus DNA
DAPI	4′,6-diamidino-2-phenylindole
DMEM	Dulbecco’s Modified Eagle Media
DMSO	dimethyl sulfoxide
DPBS	Dulbecco’s phosphate-buffered saline
EDTA	ethylenediaminetetraacetic acid
ICD	induced circular dichroism
ICG	indocyanine green
OY	oxazole yellow
PS	photosensitizing agent
ROS	reactive oxygen species
TAE	tris-acetate-EDTA
TO	thiazole orange
