# Supplementary material for: DNA Photocleavage in the Near-Infrared Wavelength Range by 2-Quinolinium Dicarbocyanine Dyes
Source: Molecules. 2020 Jun 25;25(12):2926. doi: 10.3390/molecules25122926 (PMC7355653; doi:10.3390/molecules25122926)
Supplement: Supplementary file 1 [file molecules-25-02926-s001.pdf]

Supplementary materials

DNA Photocleavage in the Near-Infrared Wavelength Range by 2-Quinolinium Dicarbocyanine Dyes

Effibe O. Ahoulou [1], Kaitlyn K. Drinkard [1], Kanchan Basnet [1], Anna St. Lorenz [3], Oleh Taratula [3], Maged Henary [1,2]* and Kathryn B. Grant [1]*

[1]Department of Chemistry, Georgia State University, [2]Center for Diagnostics and Therapeutics, Atlanta, GA 30303, United States; [3]Department of Pharmaceutical Sciences, College of Pharmacy, Oregon State University, Portland, OR 97201, United States

<u>**Contents**</u>:

Fig. S1. DNA photocleavage at 741 nm in the presence of chemical additives…….................. S2

Fig. S2. Toxicity of dye **4** against ES2 cells under dark conditions……………………….….. S3

Fig. S3. ^{1}H NMR spectrum of dye **3** ……………………………………………………… S4

Fig. S4. ^{1}H NMR spectrum of dye **4** ……………………………………………..…….. S5

Reference ……………………………………………….…………………………..…….. S6

A) Dye 4: sodium benzoate

Lane:	1	2	3

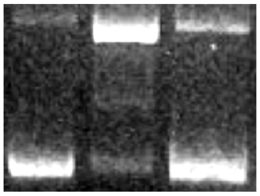

Dye	-	4	4
hν:	-	+	+
SB	-	-	+
Yield (%):	8±3	94±9	22±10

B) Dye 4: EDTA

Lane:	1	2	3

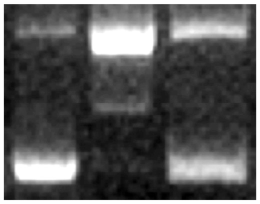

Dye	-	4	4
hν:	-	+	+
EDTA	-	-	+
Yield (%):	10±3	89±16	25±11

C) Dye 4: Catalase

Lane:	1	2	3

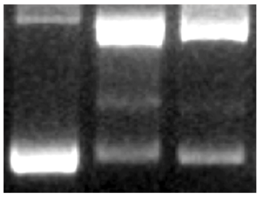

Dye	-	4	4
hν:	-	+	+
Catalase	-	-	+
Yield (%):	5±1	66±14	47±16

D) Dye 4: D_2O

Lane:	1	2	3

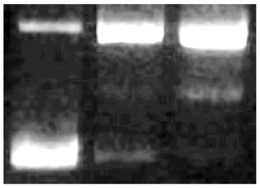

Dye	-	4	4
hν:	-	+	+
D_2O	+	+	-
Yield (%):	15±1	87±1	97±1

Figure S1. Representative agarose gels comparing levels of cyanine dye-sensitized photocleavage of pUC19 plasmid DNA generated in the absence and presence of: (**A**) 100 mM sodium benzoate; (**B**) 100 mM ethylenediaminetetraacetate (EDTA); (**C**) 100 U/μL catalase; (**D**) 72% D_2O *(v/v)* (741 nm hν for 60 min at 22 °C). All reactions contained 10 mM sodium phosphate buffer pH 7.0, 24 μM of dye **4** and 38 μM bp DNA. Yields are averaged over two to three trials. Errors represent standard deviation. Abbreviations: **L** = linear; **N** = nicked; **S** = supercoiled.

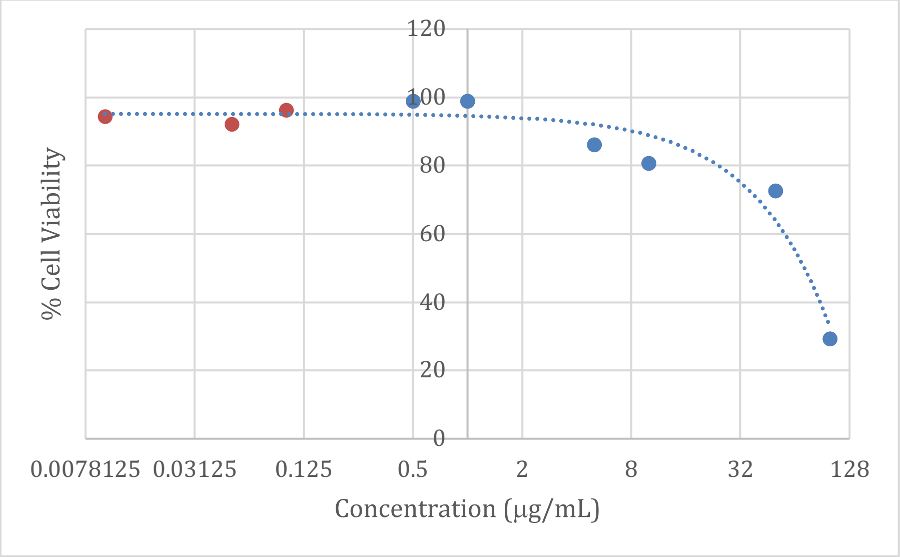

Figure S2. Viability of ES2 cells incubated for 24 h under dark conditions with different concentrations of dye **4**. ES2 cells were plated in 96-well plates at a density of 10×10^3 cells/well and cultured for 24 h. After that, the cells were incubated in the dark for 24 h in complete DMEM media containing different concentrations of dye **4** (0.01 - 100 µg/mL) dissolved in DMSO (<1%). The dye-containing media was then removed, and the cells were rinsed with DPBS and cultured for 24 h in complete DMEM growth medium prior to viability measurements with Calcein AM as previously described.[1]

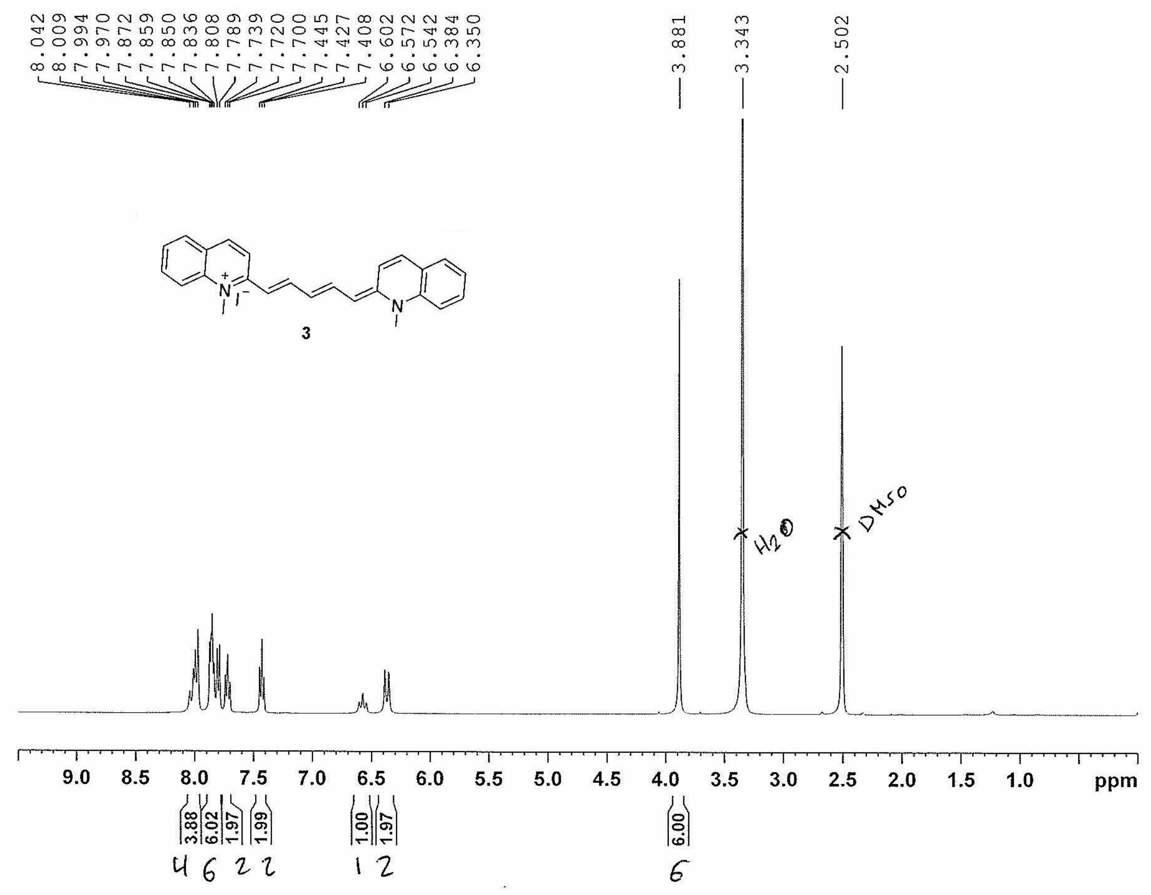

Figure S3. ^{1}H NMR spectrum of dye **3**.

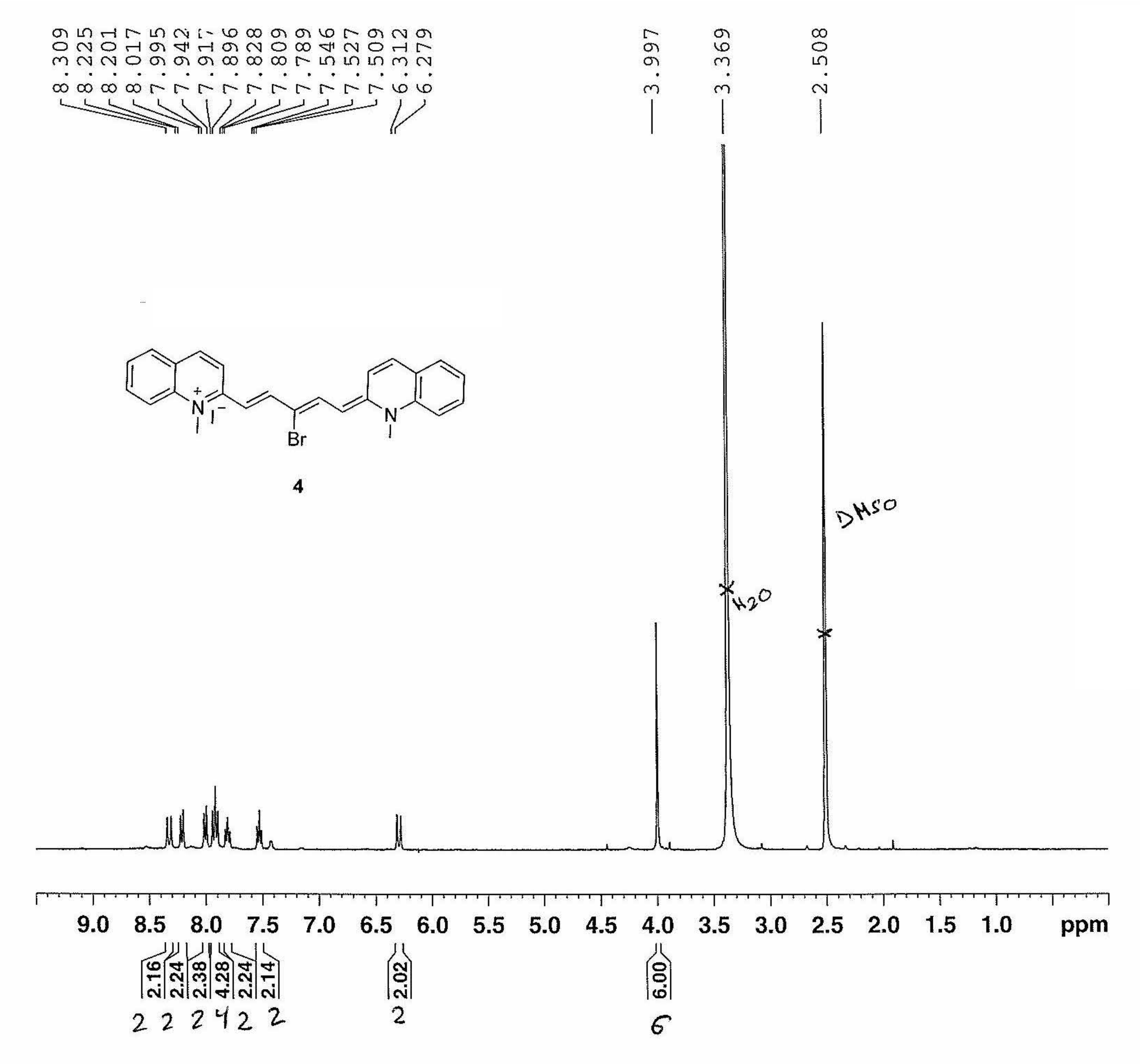

Figure S4. ^{1}H NMR spectrum of dye **4**.

Reference

1. Dani, R. K.; Schumann, C.; Taratula, O.; Taratula, O., Temperature-tunable iron oxide nanoparticles for remote-controlled drug release. *AAPS PharmSciTech* **2014,** 15, (4), 963-972.
